# Supplementary material for: Impact of combined medication payment management policies on population health performance
Source: PLoS One. 2026 Jan 9;21(1):e0330057. doi: 10.1371/journal.pone.0330057 (PMC12788656; doi:10.1371/journal.pone.0330057)
Supplement: Attachment — Submitted filename: Response Letter.docx [file pone.0330057.s001.docx]

**Response Letter**

Dear Dr. Jim Lovell Santos Gonzales and Editorial Team,

Thank you for reviewing our manuscript. We have fully addressed the requested revisions regarding ethical compliance as follows:

1. **Human Participants Research Checklist** has been completed and uploaded as a supplemental file. The study utilizes de-identified secondary data from the China Family Panel Studies (CFPS) without active participant recruitment, rendering prospective recruitment components not applicable. For the retrospective analysis, we confirm data access occurred in January 2025 using fully anonymized public datasets with no access to identifiable participant information, as reported in Methods Section 3.1.1 (Lines 185-195).
2. **Participant consent documentation** has been added to Section 3.1.1 (Lines188-191):"Written informed consent was obtained for all original CFPS data collection through a tiered process: respondents aged ≥16 years provided personal signatures, while those aged ≤15 years had consent formally granted by legal guardians. The standardized consent form template is publicly accessible via the CFPS website."
3. **Institutional ethics approval** is explicitly stated in Section 3.1.1 (Line 187-188):"This research employs data collected under the ongoing ethical oversight of Peking University's Biomedical Ethics Committee (IRB No. IRB00001052-14010, valid through 2026)."
   Submitted files include:

- Revised manuscript with tracked changes
- Completed PLOS Human Participants Checklist
- Ethics approval documentation statement
- This response letter

We confirm all revisions have been implemented in both the manuscript text and submission system ethics statement field. The PDF has been rebuilt and approved for submission.

Sincerely,

Yun Yang
