# Supplementary material for: Impact of combined medication payment management policies on population health performance
Source: PLoS One. 2026 Jan 9;21(1):e0330057. doi: 10.1371/journal.pone.0330057 (PMC12788656; doi:10.1371/journal.pone.0330057)
Supplement: Attachment — Submitted filename: Response to Reviewers.docx [file pone.0330057.s002.docx]

**Response to the Academic Editor and Reviewers**

**Dear Dr. Alexandre Morais Nunes and Reviewers,**

We are truly grateful for the opportunity to revise our manuscript (ID: PONE-D-25-40553), entitled “Impact of Combined Medication Payment Management Policies on Population Health Performance”. We sincerely appreciate the editors and reviewers for their precious time and insightful comments, which are immensely helpful in improving the quality and rigor of our work. We have carefully considered all the comments and have made extensive revisions to the manuscript accordingly. Point-by-point responses to the comments are provided below.

In addition to the specific revisions made in response to the reviewers' comments, we have also performed a comprehensive language polishing throughout the manuscript to enhance its overall fluency, clarity, and adherence to academic writing standards.

This is to clarify that every line number referenced in this letter pertains to the tracked-changes version of our manuscript (attached as ‘Revised Manuscript with Track Changes’), which includes all our revisions highlighted for your convenience.

Response to Journal Requirements:

Comment1: *Please ensure that your manuscript meets PLOS ONE's style requirements, including those for file naming.*

Response1: We have thoroughly reformatted the manuscript according to the PLOS ONE style templates. All files have been renamed according to the journal's requirements.

Comment2: *Thank you for stating the following financial disclosure... Please state what role the funders took in the study... Please include this amended Role of Funder statement in your cover letter.*

Response2: As instructed, we have added the following statement to the Funding of our cover letter:“The funders had no role in study design, data collection and analysis, decision to publish, or preparation of the manuscript.”

Comment3: *Your ethics statement should only appear in the Methods section of your manuscript. If your ethics statement is written in any section besides the Methods, please move it to the Methods section and delete it from any other section.*

Response3: We appreciate the editor's guidance on this matter. To adhere strictly to the journal's style requirements, we have made the following revisions: Firstly, we have renamed the section "3. Research Design" to "3. Materials and Methods" for better alignment with standard academic formatting. Secondly, and most importantly, we have ensured that the ethics statement is now contained only within this Methods section, specifically in subsection '3.3.1 Data Sources'. We have performed a thorough check to confirm its complete removal from any other section. The updated ethics statement in the manuscript is:

"This research uses de-identified public data from the CFPS, accessed in January 2025. The analysis complies with all ethical standards for secondary data analysis. The original CFPS project was approved by the Biomedical Ethics Committee of Peking University (IRB No. IRB00001052-14010). Written informed consent was obtained from all original participants: respondents aged ≥16 years signed independently, and for those aged ≤15 years, consent was provided through their guardians. The data were accessed from fully anonymized repositories with no identifiable information. (Consent form template: CFPS Website)"

**These changes can be found in:Page20,line376-386**

Comment4: *If the reviewer comments include a recommendation to cite specific previously published works, please review and evaluate these publications to determine whether they are relevant and should be cited. There is no requirement to cite these works unless the editor has indicated otherwise.*

Response4: We have carefully reviewed the literature in our field and have ensured that all citations are relevant and appropriate. No specific citation recommendations were made by the reviewers in this round.

Response to Reviewer #1:

**Comment1:***However, the non-significant reduction in out-of-pocket costs (Table 8) needs further discussion to clarify its implications for policy effectiveness. Has the statistical analysis been performed appropriately and rigorously.*

**Response1:** We are deeply grateful to the reviewer for this critical insight, which has allowed us to significantly strengthen the interpretation of our results. We have extensively revised the Discussion and Conclusion sections to provide a deeper, more nuanced analysis of this key finding.

We have thoroughly addressed this issue in the revised manuscript, particularly in the Discussion section (Section 4.2) and the Research Conclusions (Section 7.1). Specifically, we explain that the lack of statistical significance in out-of-pocket reduction does not imply policy ineffectiveness but reflects the initial prioritization of resource reallocation within the healthcare system. During the early stages of policy implementation, the savings from volume-based procurement were primarily redirected to cover the costs of innovative drugs included through the national negotiation mechanism. This reallocation initially benefited the healthcare supply side, resulting in a time lag before patients directly experienced reduced financial burdens.

Furthermore, in Section 7.2.3, we propose the recommendation to "Accelerate the Transmission of Policy Savings to Patient Financial Protection." The future policy direction should focus on achieving a natural reduction in patients' financial burdens through structural reforms, with the core rationale being to position the reform objective as promoting the structural optimization and efficiency enhancement of total medical expenditures. It is essential to maintain the combined strategy of "centralized procurement" and "national price negotiations." By leveraging the synergy of these two instruments, the growth rate of total medical expenditures can be effectively guided toward a rational level or even a decline. Thus, the alleviation of patients' out-of-pocket costs will become an endogenous effect arising from the improved overall efficiency of the system, rather than an outcome imposed by external intervention. Consequently, the non-significant reduction in out-of-pocket spending can be reframed as a strategic signal that the policy initially prioritized system-level optimization, rather than indicating a lack of effectiveness. We thank the reviewer for this insightful comment, which has significantly deepened our discussion.

**These changes can be found in:**

Discussion Section 4.2: [Page 27, Lines 456-468]

Conclusion Section 7.1: [Page 45, Lines784-794]

Advise Section 7.2.2: [Page 48, Lines846-858]

**Comment2:** *More detail on the 30-month bandwidth and polynomial order selection is needed. Justify bandwidth and polynomial order; consider narrower bandwidth sensitivity tests.*

**Response2:** We sincerely thank the reviewer for this critical methodological suggestion. We fully agree that transparent justification of these key parameters is essential for the robustness and credibility of our RDiT analysis. In response, we have added a comprehensive new subsection (3.2.2 Bandwidth and Polynomial Order) to the Methods section of our manuscript to provide a detailed, step-by-step explanation of our selection process.

1. Bandwidth Selection (h=30):

We fully acknowledge that the Regression Discontinuity in Time design is fundamentally intended to estimate a Local Average Treatment Effect (LATE) proximate to the cutoff point. Our choice of a 30-month bandwidth was driven by the imperative to ensure the statistical reliability, precision, and robustness of our estimate of this local effect.

Our decision was guided by the following principled considerations:

Insufficient Statistical Power at Narrower Bandwidths: The data-driven MSE-optimal bandwidth (h=12.8 months) yielded a critically small effective sample size to the right of the cutoff (N=1,191). This severely limited sample would result in high estimation variance and unacceptably low statistical power, greatly increasing the risk of a Type II error (failing to detect a true effect). While a bandwidth of h=24 months provided a statistically significant result, its sample sizestill posed concerns regarding estimation precision compared to wider bandwidths.

Optimizing the Bias-Variance Trade-off for Robustness: Our comprehensive sensitivity analysis (Table 11) reveals a key insight: the estimated treatment effect is highly stable in magnitude and remains statistically significant across a range of bandwidths from 24 to 42 months. This stability is crucial—it indicates that expanding the bandwidth from h=24 to h=30 does not introduce substantial bias , but it meaningfully reduces estimation variance by incorporating more data. Our choice of h=30 is therefore positioned within this plateau of stability, optimally balancing the fundamental bias-variance trade-off to produce the most reliable and robust estimate of the LATE.

Theoretical Coherence with Policy Implementation Dynamics: We concur that RDD estimates a local effect. A 30-month window (2.5 years) represents a theoretically justifiable "local" period for this policy context. The complex mechanisms of volume-based procurement and national negotiations including hospital formulary adjustments, shifts in prescribing practices, and patient uptake of new drug regimens unfold over a period of months and years. A bandwidth of 30 months ensures we capture these stabilized short-to-medium-run effects without being confounded by transient initial fluctuations or very long-term trends unrelated to the core policy shock.

In conclusion, we selected h=30 is to ensure our estimate of the local effect is statistically powerful, precise, and robust. This bandwidth provides a sufficient local neighborhood around the cutoff to overcome data limitations while firmly remaining within the range where the local linearity assumption is defensible and the estimates are stable.

**Table 11 Regression results for different bandwidths**

|  | (1) | (2) | (3) | (4) | (5) |
| --- | --- | --- | --- | --- | --- |
|  | h=12.8 | h=24 | h=30 | h=36 | h=42 |
| RD_Estimate | 0.020 | 0.041^***^ | 0.032^***^ | 0.044^***^ | 0.049^***^ |
|  | (0.032) | (0.010) | (0.012) | (0.012) | (0.012) |
| Eff. N (Left) | 24,584 | 28,892 | 55,608 | 55,889 | 55,889 |
| Eff. N (Right) | 1,191 | 18,392 | 18,654 | 18,654 | 18,952 |

We have included the full set of sensitivity analysis results in the new Table 11 to demonstrate this robustness.

2. Polynomial Order Selection (p=2):

To determine the appropriate polynomial order, we estimated a series of parametric (OLS) models for comparison (detailed in the new Table 13). The linear model (p=1) provided a significant positive estimate. The quadratic model (p=2) offered a substantially better model fit (as indicated by higher Adjusted R-squared and lower AIC/BIC) while yielding a positive, significant, and theoretically coherent policy effect. In contrast, the cubic model (p=3), despite a slightly better fit, produced a counterintuitive negative estimate, suggesting potential overfitting, we selected the quadratic polynomial (p=2) for our baseline nonparametric RDD specification. This choice prioritizes robust and interpretable causal inference over merely maximizing in-sample fit, ensuring our results are both statistically sound and economically meaningful.

**Table 13: Model Selection for Polynomial Order in Parametric RD Estimation**

| Variable | Linear (p=1) | Quadratic (p=2) | Cubic (p=3) |
| --- | --- | --- | --- |
| Policy Effect (D) | 0.055*** | 0.066*** | -0.020* |
|  | (0.005) | (0.007) | (0.011) |
| Adjusted R-squared | 0.0229 | 0.0235 | 0.0249 |
| Akaike (AIC) | 9883.0 | 9827.7 | 9695.2 |
| Bayesian (BIC) | 9977.3 | 9940.8 | 9827.3 |
| Observations | 92,097 | 92,097 | 92,097 |

We believe these substantial additions to the methodology section directly address the reviewer's concern and significantly strengthen the transparency and rigor of our analysis.

**These changes can be found in:**

3.2.2bandwidth and polynomial order [Page 18-19, Lines 335-363]

 Bandwidth Selection (h=30):t [Page 30, Lines 498-508]

Polynomial Order Selection (p=2): [Page31-32, Lines 533-549]

**Comment3:** *Addressing potential multicollinearity among control variables (e.g., GDP per capita) would strengthen the analysis.*

**Response3:** We thank the reviewer for raising this important point regarding the potential for multicollinearity, which is crucial for ensuring the precision and stability of our coefficient estimates. We have thoroughly addressed this concern in the revised manuscript.

We recognized that variables such as *GDP per capita*, *disposable income*, and *consumption expenditure* are often highly correlated, as they all capture similar dimensions of regional economic development. To definitively diagnose and resolve this issue, we conducted a formal Variance Inflation Factor (VIF) test on our full set of initial control variables. The results (now presented in the new Table 5 of the manuscript) confirmed significant multicollinearity, with VIF values for Provincial GDP per capita, Provincial disposable income and Provincial consumption expenditure per capita exceeding acceptable thresholds.

**Table 5 VIF(before)**

|  | VIF |
| --- | --- |
| Urban | 1.08 |
| Drink | 1.01 |
| Spouse | 1.01 |
| Provincial GDP per capita of all residents | 18.07 |
| Provincial disposable income per capita of all residents | 51.46 |
| Provincial consumption expenditure per capita of all residents | 32.33 |
| Number of health care institutions in the province | 5.21 |
| Number of practicing physicians in the province | 5.11 |
| Mean VIF | 12.49 |

**Table5 VIF(after)**

|  | VIF |
| --- | --- |
| Urban | 1.08 |
| Drink | 1.01 |
| Spouse | 1.01 |
| Provincial disposable income per capita of all residents | 2.09 |
| Number of health care institutions in the province | 4.58 |
| Number of practicing physicians in the province | 4.47 |
| Mean VIF | 4.01 |

In response, we removed the two variables (Provincial GDP per capita and Provincial consumption expenditure per capita) from our model. We retained Provincial disposable income per capita as our primary measure of regional economic conditions, as it is the most direct indicator of residents' economic capacity for healthcare spending. We then re-ran the VIF test on the refined set of control variables. The results, reported in the updated Table 5, demonstrate that all remaining VIF values are well below the common critical value of 10, and the mean VIF is a low 4.01. This confirms that severe multicollinearity is no longer a concern in our final model specification.

Most importantly, we re-estimated all our main regression models (e.g., Tables7-12) using this refined set of control variables. The key findings regarding the policy's impact on health performance, effectiveness, and regional heterogeneity remain qualitatively unchanged and statistically robust. This confirms that our core conclusions are not sensitive to the specific set of economic control variables and are based on a well-specified model.

We believe these steps have significantly strengthened the methodological rigor of our analysis.

**These changes can be found in:**

3.3.4 Multicollinearity Test [Page 22-23, Lines 392-400]

**Comment4:** *Minor improvements, like reducing repetition of “health performance” and clarifying jargon (e.g., “vacating the cage and exchanging the bird”), would improve readability.*

**Response 4:** We thank the reviewer for these suggestions. To convey the precise dimensions of “health performance,” we now employ a richer, context-specific vocabulary instead of the generic phrase. In parallel, the culture-bound metaphor “vacating the cage and exchanging the bird” has been replaced with the explicit description “phasing out low-value or inefficient products while introducing clinically superior and cost-effective alternatives.”

These changes have been made throughout the manuscript to ensure it is accessible and clear to a broad readership.

**These changes can be found in:**

Introduction [Page 4, Lines 52-53]

**Comment5:** *Support import/export claims with data.*

**Response5:** We thank the reviewer for this suggestion. In response, we have added descriptive statistics and a visual analysis to strengthen the empirical support for our claims.

Our revisions are twofold:

1.Incorporation of Descriptive Statistics: We have now integrated statistics into the narrative of Sections 6.3.1 We explicitly reference the macro-level trends, noting the changes in import and export values in the periods immediately before and after policy implementation to ground our discussion in tangible data.

2.Addition of a Visual Counterfactual Analysis: As prominently suggested by the reviewer, we have added a new figure (Fig. 8: Trends in China's Pharmaceutical Import and Export Value (2016-2022)) to Section 6.3.1. This figure is not merely a plot of raw data; it extends the pre-policy (2016-2018) trends to establish a counterfactual baseline. This allows for a direct visual comparison between what actually happened post-policy and what would have been expected in the policy's absence. The clear divergence of the actual data from the forecasted paths provides immediate and intuitive graphical evidence of the policy's effects:

For Imports: The actual values fall consistently below the extrapolated trend, visually confirming the policy's significant suppressive effect.For Exports: The actual values break decisively above the forecasted path, offering strong support for the policy-induced enhancement of export competitiveness.

|  | ln(Imports) | ln(Exports) | ln_import_counterfactual | ln_export_counterfactual |
| --- | --- | --- | --- | --- |
| 2016 | 25.6465 | 24.5464 | 25.6465 | 24.5464 |
| 2018 | 25.9413 | 24.7845 | 25.9413 | 24.7845 |
| 2020 | 26.2126 | 25.2317 | 26.2361 | 25.0225 |
| 2022 | 26.3114 | 25.2524 | 26.5309 | 25.2606 |

We believe these additions provide multifaceted and robust evidence for our investigation into the policy's impact on pharmaceutical innovation and competition, leveraging the perspective of drug trade data.

**These changes can be found in:**

6.3.1-6.3.2[Page 40-44]

**Comment6:** *Propose region-specific policies.*

**Response6:** We thank the reviewer for suggesting more region-specific policy recommendations. In response, we have substantially revised Section 7.2.3 to provide more geographically differentiated and implementable strategies.

The key improvements include:

Clearer Regional Differentiation: We now explicitly separate policies for Eastern, Central, and Western China, rather than grouping central and western regions together. This better reflects the distinct economic and healthcare contexts of these regions.

More Specific Interventions:

Eastern China :Set pilot intra-/extra-directory coordinated payment mechanisms to help high-income populations access non-listed innovative drugs through commercial insurance tailored to differentiated needs.

Central China: The policy focus should shift towards translating the "cost-reduction effect" of centralized procurement reform into tangible "quality-improvement outcomes. Pivotal to this effort are ensuring the stable integration of high-quality pharmaceuticals into the clinical supply system and rigorously standardizing prescribing behaviors to enhance the rationality of clinical medication.

Western China:fiscal transfers should be increased to expand the scope of collective procurement, ensure the supply of basic medicines, and meet rising demand for rational medication.

The revised version more accurately matches policy interventions to each region's specific challenges and development level.

**These changes can be found in:**

7.2.2 [Page 47, Lines 832-845]

Response to Reviewer #2:

**Comment1:** *It is suggested that there be a more detailed description regarding the selection of specific bandwidths and polynomial orders in the breakpoint regression.*

**Response1:** We sincerely thank the reviewer for this critical methodological suggestion. We fully agree that transparent justification of these key parameters is essential for the robustness and credibility of our RDiT analysis. In response, we have added a comprehensive new subsection (3.2.2 Bandwidth and Polynomial Order) to the Methods section of our manuscript to provide a detailed, step-by-step explanation of our selection process.

1. Bandwidth Selection (h=30):

We fully acknowledge that the Regression Discontinuity in Time design is fundamentally intended to estimate a Local Average Treatment Effect (LATE) proximate to the cutoff point. Our choice of a 30-month bandwidth was driven by the imperative to ensure the statistical reliability, precision, and robustness of our estimate of this local effect.

Our decision was guided by the following principled considerations:

Insufficient Statistical Power at Narrower Bandwidths: The data-driven MSE-optimal bandwidth (h=12.8 months) yielded a critically small effective sample size to the right of the cutoff (N=1,191). This severely limited sample would result in high estimation variance and unacceptably low statistical power, greatly increasing the risk of a Type II error (failing to detect a true effect). While a bandwidth of h=24 months provided a statistically significant result, its sample sizestill posed concerns regarding estimation precision compared to wider bandwidths.

Optimizing the Bias-Variance Trade-off for Robustness: Our comprehensive sensitivity analysis (Table 11) reveals a key insight: the estimated treatment effect is highly stable in magnitude and remains statistically significant across a range of bandwidths from 24 to 42 months. This stability is crucial—it indicates that expanding the bandwidth from h=24 to h=30 does not introduce substantial bias , but it meaningfully reduces estimation variance by incorporating more data. Our choice of h=30 is therefore positioned within this plateau of stability, optimally balancing the fundamental bias-variance trade-off to produce the most reliable and robust estimate of the LATE.

Theoretical Coherence with Policy Implementation Dynamics: We concur that RDD estimates a local effect. A 30-month window (2.5 years) represents a theoretically justifiable "local" period for this policy context. The complex mechanisms of volume-based procurement and national negotiations including hospital formulary adjustments, shifts in prescribing practices, and patient uptake of new drug regimens unfold over a period of months and years. A bandwidth of 30 months ensures we capture these stabilized short-to-medium-run effects without being confounded by transient initial fluctuations or very long-term trends unrelated to the core policy shock.

In conclusion, we selected h=30 is to ensure our estimate of the local effect is statistically powerful, precise, and robust. This bandwidth provides a sufficient local neighborhood around the cutoff to overcome data limitations while firmly remaining within the range where the local linearity assumption is defensible and the estimates are stable.

**Table 11 Regression results for different bandwidths**

|  | (1) | (2) | (3) | (4) | (5) |
| --- | --- | --- | --- | --- | --- |
|  | h=12.8 | h=24 | h=30 | h=36 | h=42 |
| RD_Estimate | 0.020 | 0.041^***^ | 0.032^***^ | 0.044^***^ | 0.049^***^ |
|  | (0.032) | (0.010) | (0.012) | (0.012) | (0.012) |
| Eff. N (Left) | 24,584 | 28,892 | 55,608 | 55,889 | 55,889 |
| Eff. N (Right) | 1,191 | 18,392 | 18,654 | 18,654 | 18,952 |

We have included the full set of sensitivity analysis results in the new Table 11 to demonstrate this robustness.

2. Polynomial Order Selection (p=2):

To determine the appropriate polynomial order, we estimated a series of parametric (OLS) models for comparison (detailed in the new Table 13). The linear model (p=1) provided a significant positive estimate. The quadratic model (p=2) offered a substantially better model fit (as indicated by higher Adjusted R-squared and lower AIC/BIC) while yielding a positive, significant, and theoretically coherent policy effect. In contrast, the cubic model (p=3), despite a slightly better fit, produced a counterintuitive negative estimate, suggesting potential overfitting, we selected the quadratic polynomial (p=2) for our baseline nonparametric RDD specification. This choice prioritizes robust and interpretable causal inference over merely maximizing in-sample fit, ensuring our results are both statistically sound and economically meaningful.

**Table 13: Model Selection for Polynomial Order in Parametric RD Estimation**

| Variable | Linear (p=1) | Quadratic (p=2) | Cubic (p=3) |
| --- | --- | --- | --- |
| Policy Effect (D) | 0.055*** | 0.066*** | -0.020* |
|  | (0.005) | (0.007) | (0.011) |
| Adjusted R-squared | 0.0229 | 0.0235 | 0.0249 |
| Akaike (AIC) | 9883.0 | 9827.7 | 9695.2 |
| Bayesian (BIC) | 9977.3 | 9940.8 | 9827.3 |
| Observations | 92,097 | 92,097 | 92,097 |

We believe these substantial additions to the methodology section directly address the reviewer's concern and significantly strengthen the transparency and rigor of our analysis.

**These changes can be found in:**

3.2.2bandwidth and polynomial order [Page 18-19, Lines 335-363]

 Bandwidth Selection (h=30):t [Page 30, Lines 498-508]

Polynomial Order Selection (p=2): [Page31-32, Lines 533-549]

**Comment2:** *The discussion be included regarding the potential limitations of the RDiT approach.*

**Response2** We thank the reviewer for this important suggestion. We agree that a discussion of the limitations of the Regression Discontinuity in Timedesign is crucial for a comprehensive interpretation of our findings.

In direct response to this comment, we have now added a new subsection (7.4 Limitations and Future Research) to the manuscript. In this section, we explicitly acknowledge and discuss two key limitations inherent to our empirical strategy:1.The potential for confounding events coinciding with the 2019 policy implementation timepoint, despite our efforts to control for observable factors.2.The study's focus on short-to-medium-term effects, leaving the long-term sustainability of the observed outcomes as an open question for future research.

We also outline directions for future studies, suggesting the use of longer time series and alternative methodological approaches to validate and extend our results.

We believe this addition provides a balanced and critical perspective on our research, strengthening the paper's scholarly contribution.

**These changes can be found in:**

7.4 Limitations and Future Research [Page 50-51, Lines 903-913]

**Comment3:** *The study is based on CFPS data (2016–2022), which is representative, and applies entropy weighting to construct composite health performance indicators. The approach is valid, integrating self-assessment of health, sudden and chronic illnesses, and expense-related measures. The index description is brief. Without additional robustness tests, the validity of the results and conclusions is called into question. It is suggested to conduct complementary or sensitivity analyses to observe the consistency of the results and evidence.*

**Response3:** We sincerely thank the reviewer for this valuable suggestion. We fully agree that a detailed description of the key variable and robustness checks are crucial for ensuring the reliability of our findings. In response, we have made the following two major enhancements in the revised manuscript:

Enhanced Description of the Composite Health Index: We have significantly expanded the description of the index construction using the entropy weight method in Section 3.1, "Resident Health Performance Indicators and Measurement." The revised text now provides a clearer rationale for the method, along with explanations for data treatments like the logarithmic transformation of expenditure variables, ensuring full transparency.

Added Robustness Check using Principal Component Analysis (PCA): Following the reviewer's suggestion, we conducted a crucial robustness test by reconstructing the health performance index using Principal Component Analysis (PCA). The analysis showed that the first three principal components explained 77.55% of the total variance. A composite PCA score was generated using the variance contribution rates as weights. This PCA score demonstrated a very high correlation (r = 0.9406) with the original entropy weight score. We then re-estimated our main Regression Discontinuity in Time model using this alternative index. The results (now presented in Table 14 of the manuscript) confirm that the estimated policy effect remains significantly positive at the 1% level, aligning perfectly in direction and significance with our primary results based on the entropy weight method. This provides strong evidence that our core conclusion is robust to the choice of index construction methodology.

We believe these revisions have largely addressed the reviewer’s concerns and have appreciably enhanced the methodological rigor and persuasive power of our study

**Table 14: Comparison of Different Health Performance Index Construction Methods**

|  | (1) | (2) |
| --- | --- | --- |
|  | PCA | entropy weight method |
| RD | 0.164*** | 0.034*** |
|  | (0.038) | (0.010) |
| Obs | 92096 | 92097 |

**These changes can be found in:**

3.1.1 Indicator Design[Page 11-12, Lines213-232]

5.5 Index Reconstruction based on Principal Component Analysis [Page 32-34, Lines550-574]

**Comment4:** *The causal inferences exceed the gathered evidence, especially when associating long-term industrial innovation changes with the implementation of short-term policies. It is suggested that causal inferences be moderated; they should be directly related to the observed evidence. The analysis indicates significant correlations and plausible mechanisms, but it must clearly distinguish between the immediate effects of policies and the hypothetical long-term trajectories./*

*While the empirical evidence attests to efficiency gains and moderate improvements in health outcomes, the recommendations are presented as mere claims about long-term industrial and innovation transformation, which were not directly measured in the dataset. It is suggested that policy recommendations be directly aligned with empirical evidence. The recommendations should be framed as prospective implications, clearly differentiated from the conclusions supported by empirical analysis*

**Response4:** We are profoundly grateful to the reviewer for this crucial insight, which has guided us in significantly refining the interpretation and presentation of our findings to ensure strict alignment between our claims and the gathered evidence. In direct response, we have undertaken a comprehensive revision of Chapter 7 (Research Conclusion and Policy Recommendations) to meticulously moderate our causal language and clearly differentiate between empirically supported conclusions and prospective implications. The specific modifications are as follows:

Substantial Revisions to Section 7.1 (Research Conclusions):

We have meticulously moderated the language regarding long-term industrial transformation. Specifically, we now frame the observed increases in R&D and shifts in trade patterns as establishing a "promising foundation" or "providing a necessary catalytic condition" for potential long-term upgrading, rather than as direct proof of its full realization. Crucially, we added an explicit caveat: "It is important to note that these findings primarily reflect short-to-medium term effects following policy implementation. While the observed increase in R&D and shifts in trade patterns provide a promising foundation, their long-term sustainability and ultimate impact on industrial transformation require further longitudinal study." This clearly bounds our conclusions within the timeframe supported by our data.

Refinement of Section 7.2 (Policy Recommendations Based on Empirical Evidence):

We have systematically ensured that all policy recommendations are directly derived from our short-to-medium term empirical findings. For instance:

The recommendation to "Establishing a quality-benefit two-dimensional evaluation system" is directly tied to our findings on performance and effectiveness gains.

The proposal to "egionally differentiated supply strategies to address structural imbalances in medical resources" directly stems from our empirical finding of significant heterogeneity across the eastern China, central China, and western China.

The suggestion to "Accelerate the Transmission of Policy Savings to Patient Financial Protection" is a direct response to the non-significant reduction in out-of-pocket costs.

We have removed or reframed recommendations that were speculative and not directly anchored in the evidence presented.

Addition of a New Section 7.3 (Prospective Implications for Long-Term Development):

As suggested by the reviewer, we created this entirely new section to explicitly house forward-looking discussions that extend beyond our direct empirical evidence. Here, we clearly frame the long-term significance of our short-term findings as "prospective implications" and "forward-looking implications derived from early signals." We state: "The short-term empirical findings of this study, particularly the significant increase in pharmaceutical R&D investment and output, offer valuable insights for long-term policy planning... It is crucial to note that these are forward-looking implications derived from early signals; their realization requires continuous policy commitment and warrants further long-term investigation." This creates a clear demarcation between our empirical conclusions (Section 7.1) and speculative yet valuable future-oriented discussion (Section 7.3).

We believe these revisions have successfully addressed the reviewer's concern by ensuring that causal inferences are moderated, policy recommendations are empirically anchored, and long-term trajectories are presented explicitly as hypothetical implications rather than established conclusions. Thank you for prompting this significant improvement in the scholarly rigor of our manuscript.

**These changes can be found in:**

Chapter 7:

sections 7.1 Research Conclusions [Page 44-46]

7.2 Policy Recommendations Based on Empirical Evidence [Page 46-49]

7.3 Prospective Implications for Long-Term Development[Page 50]
